# An asymmetric approach to preserve common intervals while sorting by reversals

**DOI:** 10.1186/1748-7188-4-16

**Published:** 2009-12-30

**Authors:** Marília DV Braga, Christian Gautier, Marie-France Sagot

**Affiliations:** 1 Université de Lyon, F-69000, Lyon; Université Lyon 1; CNRS UMR5558, Inria Grenoble Rhône-Alpes, France; 2 Current address: AG Genominformatik, Technische Fakultät, Universität Bielefeld, Germany

## Abstract

**Background:**

The reversal distance and optimal sequences of reversals to transform a genome into another are useful tools to analyse evolutionary scenarios. However, the number of sequences is huge and some additional criteria should be used to obtain a more accurate analysis. One strategy is searching for sequences that respect constraints, such as the common intervals (clusters of co-localised genes). Another approach is to explore the whole space of sorting sequences, eventually grouping them into classes of equivalence. Recently both strategies started to be put together, to restrain the space to the sequences that respect constraints. In particular an algorithm has been proposed to list classes whose sorting sequences do not break the common intervals detected between the two inital genomes *A *and *B*. This approach may reduce the space of sequences and is symmetric (the result of the analysis sorting *A *into *B *can be obtained from the analysis sorting *B *into *A*).

**Results:**

We propose an alternative approach to restrain the space of sorting sequences, using progressive instead of initial detection of common intervals (the list of common intervals is updated after applying each reversal). This may reduce the space of sequences even more, but is shown to be asymmetric.

**Conclusions:**

We suggest that our method may be more realistic when the relation ancestor-descendant between the analysed genomes is clear and we apply it to do a better characterisation of the evolutionary scenario of the bacterium *Rickettsia felis *with respect to one of its ancestors.

## Background

Genomes are not static but are instead subject to continuous mutations during evolution. These mutations can be of different types and scales. Events such as single nucleotide polymorphisms (SNPs), that affect only one nucleotide at a time, are said to be small scale events, and are more frequent than large scale events [1]. The main known rearrangements or large scale events are reversals of large portions of chromosomes, insertions of new genes (usually due to duplications or horizontal transfer between species), deletions or loss of genes, transpositions of DNA fragments within a chromosome, fusions and/or fissions of chromosomes, translocations of DNA fragments between chromosomes.

Reversals are among the rearrangement events more frequently observed, specially (but not exclusively) in the evolution of prokaryotes. Most of the existing differences between six species of the *Rickettsia *bacterium thus appear to be explained by reversals [2]. Computing the reversal distance, that is, the minimum number of reversals required to transform a genome into another, and finding one optimal sequence of reversals that transforms one genome into the other are useful tools to analyse real evolutionary scenarios. When duplications are not allowed, both problems can be solved in polynomial time [3-5]. These two problems have been the topic of several works [5-8] and can be solved with the aid of some currently available softwares. One is the package GRAPPA[9] (Genome Rearrangements Analysis under Parsimony and other Phylogenetic Algorithms), that contains several programs to deal with genome rearrangements and can be downloaded at http://www.cs.unm.edu/~moret/GRAPPA/. Another is the software GRIMM[10], that contains also algorithms for multichromosomal genome rearrangements and is available online at http://grimm.ucsd.edu/GRIMM/. These programs were used in particular by Blanc et al. [2] in the analysis of the *Rickettsia *bacteria.

Other approaches are also able to find one optimal sorting sequence, but this is often insufficient to allow a proper analysis, since there are many different sequences, and taking one is not enough to evaluate the evolutionary scenario in a realistic way. In order to select a more meaningful sequence, a good strategy is to consider some biological constraints. A promising constraint to this purpose is the list of clusters of co-localised genes, which are common intervals of the genomes composed by the same genes but not necessarily in the same order and orientations [11]. A sorting sequence of reversals that does not cut any common interval detected between the two initial genomes *A *and *B *may be more accurate than a sorting sequence that does not have this property. In addition, this approach is symmetric, that is, the result of the analysis of the sequences that sort *A *into *B *can be directly obtained from the result of the analysis of the sequences that sort *B *into *A*. Several studies take common intervals in consideration when sorting by reversals [11-14].

Exploring the whole set of sequences is also an interesting strategy to analyse the evolution of the considered organisms. The first step in this direction was an algorithm that allows the enumeration of all sequences of reversals sorting one genome into another, proposed by Siepel [15]. However, since the number of sequences is usually huge, the whole set is very hard to handle and this could be as useless as finding one sequence. Bergeron et al. [16] then proposed a model to represent the sequences in a compact way, grouping them into classes of equivalence. This method allows to reduce substantially the number of elements to be handled, and an algorithm to directly enumerate all the classes was given by Braga et al. [17].

Braga et al. started to put both strategies together, that is, to construct only the classes whose sequences respect some biological constraints. The authors showed that it is possible to reduce the number of classes by selecting only those composed by sequences whose reversals do not cut any common interval initially detected [17]. In the present work, we propose a variation of this approach which, instead of initial detection, uses a progressive detection of common intervals to explore the solution space of sorting by reversals (the common intervals are recomputed after applying each reversal). We observe that this new approach is asymmetric, but relevant when the relation ancestor-descendant between the studied genomes is clear. We show that it can reduce considerably the universe of solutions. We also revise a result proposed by Braga et al. [17], when the perfect constraint is relaxed to accept some common interval breaks. The consequences of introducing this relaxation have not been deeply discussed by Braga et al., and we show that this strategy also leads to asymmetric sequences of reversals.

We applied our adapted algorithm to characterise the space of all solutions between the bacterium *Rickettsia felis *and one of its ancestors, taking into account the progressively detected common intervals. Observe that we assume that the philogeny of the studied species is known, thus in this first approach our method is not used to reconstruct philogeny. However, the assymmetry of our method could be used to infer philogeny in a next step. Approaches using the reversal distance to infer philogeny exist, such as the median problem with reversals [18] and other problems of rearrangements in multiple genomes [19]. Note that these approaches consider at least three genomes and generally consist of heuristics and approximation algorithms (the reversal median problem is proven to be NP-hard [20]).

## Methods

### Permutations, intervals and reversals

We represent the studied genomes by the list of homologous markers (usually genes or blocks of contiguous genes) between them. These markers are represented by the integers 1, 2,..., *n*, with a plus or minus sign to indicate the strand they lie on. The order and orientation of the markers of one genome in relation to the other is given by a *signed permutation π *= (*π*_1_, *π*_2_,..., *π*_*n*-1_, *π*_*n*_) of size *n *over {-*n*,..., -1, 1,..., *n*}, such that, for each value *i *from 1 to *n*, either *i *or -*i *is mandatorily present, but not both. The *identity permutation *(1, 2, 3,..., *n*) is denoted by ℐ_*n*_.

A subset of numbers *ρ *⊆ {1, 2,..., *n *- 1, *n*} is said to be an *interval *of a permutation *π *if there exist *i*, *j *∈ {1,..., *n*}, 1 ≤ *i *≤ *j *≤ *n*, such that *ρ *= {|*π*_*i*_|,|*π*_*i*+1_|,..., |*π*_*j*-1_|,|*π*_*j*_|}. Given a permutation *π *and an interval *ρ *of *π*, we can apply a *reversal *on the interval *ρ *of *π*, that is, the operation which reverses the order and flips the signs of the elements of *ρ*, denoted by *π *∘ *ρ*. If

*π *= (*π *_1_,..., *π*_*i*-1_, *π*_*i*_, *π*_*i*+1_,..., *π*_*j*-1_, *π*_*j*_, *π*_*j*+1_,..., *π*_*n*_) and *ρ *= {|*π*_*i*_|,|*π*_*i*+1_|,..., |*π*_*j*-1_|,|*π*_*j*_|}, *π *∘ *ρ *is

For example, with the permutation *π *= (-3, 2, 1, -4) and the interval *ρ *= {1, 2, 4} we have *π *∘ *ρ *= (-3, 4, -1, -2). Due to this, an interval *ρ *can also be used to denote a reversal. An *i*-*sequence *of reversals *ρ*_1_*ρ*_2_...*ρ*_*i *_is valid for a permutation *π *if *ρ*_1 _is an interval of *π*, *ρ*_2 _is an interval of *π *∘ *ρ*_1_, *ρ*_3 _is an interval of (*π *∘ *ρ*_1_) ∘ *ρ*_2_, and so on. If *ρ*_1_*ρ*_2_...*ρ*_*i *_is a valid *i*-sequence of reversals for a permutation *π*, then *π *∘ *ρ*_1_*ρ*_2_...*ρ*_*i *_denotes the consecutive application of the reversals *ρ*_1_, *ρ*_2_,...*ρ*_*i *_in the order in which they appear. We say that an *i*-sequence of reversals *ρ*_1_...*ρ*_*i *_*sorts *a permutation *π *into a permutation *π*_*T *_if *π *∘ *ρ*_1_...*ρ*_*i *_= *π*_*T*_.

The length of a shortest sequence of reversals sorting a permutation *π *into *π*_*T *_is called the *reversal distance *of *π *and *π*_*T*_, and is denoted by *d*(*π, π*_*T*_). Let *s *= *ρ*_1_*ρ*_2_...*ρ*_*i *_be a valid *i*-sequence of reversals for a permutation *π*. If *d*(*π *∘ *s*, *π_T_*) = *d*(*π, π*_*T*_) - *i*, then *s *is said to be an *optimal i*-*sequence*. Moreover, if *s *is an optimal *i*-sequence and *i *= *d*(*π*, *π*_*T*_), then *s *is simply called an *optimal sorting sequence *for *π *and *π*_*T*_. We also define the *k*-prefix of an optimal sorting sequence *s *as the sequence composed by the first *k *reversals of *s*. Observe that if *s' *is a *k*-prefix of an optimal sequence *s *sorting *p *into *π*_*T*_, then *d*(*π *∘ *s'*, *π*_*T*_) = *d*(*π*, *π*_*T*_) - *k*, that is, *s' *is an optimal *k*-sequence for *π *and *π*_*T*_. For example, if we consider two permutations *π *= (-3, 2, 1, -4) and *π*_*T *_= ℐ_4_, we have *d*(*π*, *π*_*T*_) = 4 and one optimal sorting sequence is {1, 2, 4}{1, 3, 4}{2, 3, 4}{3}, whose 1-, 2- and 3-prefixes are {1, 2, 4}, {1, 2, 4}{1, 3, 4} and {1, 2, 4}{1, 3, 4}{2, 3, 4}.

For any sequence of reversals *s *= *ρ*_1_*ρ*_2_...*ρ*_*d*-1_*ρ*_*d *_sorting a permutation *π *into a permutation *π*_*T*_, we define the inverse of *s *as *inv*(*s*) = *ρ*_*d*_*ρ*_*d*-1_...*ρ*_2_*ρ*_1_. Observe that the sequence *inv*(*s*) sorts *π*_*T *_into *π*, and, consequently, each optimal sequence sorting *π *into *π*_*T *_has an equivalent optimal sequence sorting *π*_*T *_into *π*. Due to this, the approach of sorting one genome into another by reversals is said to be symmetric.

Henceforth we will generally use simply the term sequence or *i*-sequence to refer to an optimal sequence or optimal *i*-sequence of reversals. Without loss of generality, we often omit the target permutation *π*_*T*_. In this case, *π*_*T *_corresponds to the identity permutation ℐ_*n *_= (1, 2, 3,..., *n*), where *n *is the size of the initial permutation *π*, and the notation *d*(*π*) is equivalent to *d*(*π*, ℐ_*n*_).

### Sequences of reversals and common intervals

Clusters of co-localised genes are intervals of the genomes composed by the same genes but not necessarily in the same order and orientations. These clusters are modeled as *common intervals *between two permutations *π *and *π*_*T*_, which are the intervals of *π *that are present in *π*_*T*_, but not necessarily with the same internal order and orientations. For example, the interval {1, 2, 3} is common to the permutations *π *= (-3, 2, 1, -4) and ℐ_4 _= (1, 2, 3, 4). We say that all intervals with size equal to 1 and the interval with size *n*, that comprises the entire permutation, are trivial common intervals.

Two intervals are said to *overlap *if they intersect but none is contained in the other. For example, in the permutation (-3, 2, 1, -4), the intervals {2, 3} and {1, 2, 4} overlap, while {2, 3} and {1, 2, 3} do not. A reversal *ρ *breaks an interval *θ *if *ρ *and *θ *overlap. Thus, the reversal {1, 2, 4} breaks the interval {2, 3}, while the reversal {1, 2, 3} does not. Observe that a reversal never breaks a trivial common interval. The concept of *irreducible common intervals *has been introduced by Heber and Stoye [21]. The authors showed that any common interval *θ *between two permutations *π *and *π*_*T *_has a generating chain of intervals (*γ*_1_, *γ*_2_,..., *γ*_*k*_), such that the intervals *γ*_1_, *γ*_2_,..., *γ*_*k *_are listed in lexicographic order, and, for each pair of consecutive intervals *γ*_*j*_, *γ*_*j*+1_, we have *γ*_*j *_∩ *γ*_*j*+1_≠ ∅. A *reducible common interval *is an interval whose generating chain has length at least two, otherwise the common interval is irreducible. For example, the generating chain of the reducible common interval {1, 2, 3} between the permutations (-3, 2, 1, -4) and ℐ_4 _is ({1, 2}, {2, 3}) (the intervals {1, 2} and {2, 3} are irreducible). Testing whether a reversal breaks an irreducible common interval is sufficient to determine whether it breaks a common interval.

**Proposition 1 ***A reversal ρ breaks a reducible interval θ, if, and only if, breaks at least one irreducible interval in the chain that generates θ*.

*Proof*. It is easy to see that breaking an irreducible interval in the chain that generates a reducible interval *θ *also breaks *θ*. Since each pair of consecutive irreducible intervals in the chain that generates *θ *have a non-empty intersection, breaking *θ *breaks at least one irreducible interval in the chain that generates *θ*.    □

As a consequence of Proposition 1, if *ρ *does not break any irreducible interval between two permutations *π *and *π*_*T*_, then *ρ *does not break any reducible interval between *π *and *π*_*T *_as well. While the number of common intervals is bounded by *n*^2^, the number of irreducible common intervals is bounded by *n *[21], where *n *is the size of the input permutations.

**Figure 1:**
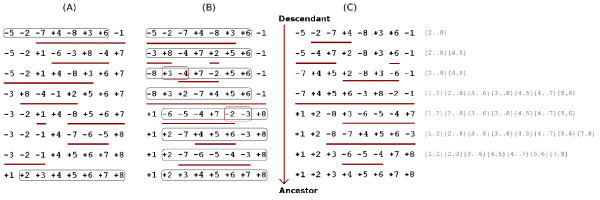
**Different approaches to select an optimal sorting sequence**. The permutations (-5, -2, -7, 4, -8, 3, 6, -1) and ℐ_8 _have only one initially detected non-trivial irreducible common interval, which is {2,..., 8}. **(A) **A sequence of reversals that sorts the permutation, but does not preserve the initially detected common interval. **(B) **A sequence of reversals that is a perfect sorting sequence (preserves the initially detected common interval), but does not preserve the new common intervals that appear during the sorting process (such as {3, 4} and {2, 3}). **(C) **A progressive perfect sequence that sorts the descendant permutation (-5, -2, -7, 4, -8, 3, 6, -1) without breaking the progressively detected irreducible common intervals (listed on the right side).

Common intervals between genomes have been the topic of several studies [11-14]. Nevertheless, in the comparison of two permutations, the detection of common intervals is usually done at the beginning of the analysis, an approach that we call *initial detection of common intervals*. An optimal sequence of reversals sorting a permutation *π *into *π*_*T *_that does not break any (irreducible) common interval initially detected between *π *and *π*_*T *_is called a *perfect sorting sequence*. Figure 1 shows a non-perfect (A) and a perfect (B) sorting sequence. We observe that the perfect sorting sequences are symmetric with respect to the initially detected common intervals. In other words, given two permutations *π *and *π*_*T*_, any perfect sequence of reversals *s *that sorts *π *into *π*_*T *_has an equivalent perfect sorting sequence *s' *that sorts *π*_*T *_into *π *: *s' *= *inv*(*s*).

In this approach, however, the new common intervals that could appear between an intermediary permutation, after applying some reversals to the initial permutation, and the target permutation, are not considered. Thus, if a common interval appears between an intermediary permutation and the target permutation, there is no constraint on the selection of a reversal that breaks this new interval (see Figure 1(B)). Alternatively to the initial detection, in this work we propose the *progressive detection of common intervals*, that consists in updating the list of (irreducible) common intervals between the permutations after each reversal. An optimal sorting sequence that does not break the progressively detected irreducible common intervals is called *progressive perfect sorting sequence*. Figure 1(C) shows an example of this approach.

Differently from the perfect sorting sequences, the progressive perfect sorting sequences are asymmetric, that is, inverting a progressive perfect sorting sequence that sorts a first into a second permutation generally does not result in a progressive perfect sorting sequence that sorts the second permutation into the first. An example is given in Figure 1(C). Observe that, applying the last reversal {4, 5, 6} on the permutation (1, 2, 3, 4, 5, 6, 7, 8) results in the permutation (1, 2, 3, -6, -5, -4, 7, 8), that has the common interval {4, 7, 8} with respect to the permutation (-5, -2, -7, 4, -8, 3, 6, -1). The reversal {3,..., 7} (the third from bottom to top in Figure 1(C)) overlaps with {4, 7, 8}, thus inverting the progressive perfect sequence of reversals {2, 4, 7}, {4, 5, 7}, {6}, {2, 3, 6, 8}, {1,..., 8}, {3,..., 7}, {3,..., 8}, {4, 5, 6} that sorts (-5, -2, -7, 4, -8, 3, 6, -1) into (1, 2, 3, 4, 5, 6, 7, 8) does not result in a progressive perfect sequence of reversals that sorts (1, 2, 3, 4, 5, 6, 7, 8) into (-5, -2, -7, 4, -8, 3, 6, -1).

When we compare current species, it is not possible to determine a direction to the analysis. In this case, considering common intervals that appear in intermediary states is meaningless and a symmetric approach is more adequate. Symmetry is thus an advantage that supports the initial detection of common intervals in many applications. We suggest however that, when the relation ancestor-descendant between the analysed genomes is clear, the progressive detection of common intervals may be more realistic than the initial detection of common intervals. In this case, the analysis should be done from the descendant to the ancestor, since the objective is to regroup intervals that may have existed in a past time.

### Common intervals in the analysis of the space of optimal sorting sequences

Finding one optimal sequence of reversals that sorts a permutation into another is only one part of the information required to analyse an evolutionary scenario, even when we get a sequence that does not break the common intervals. The number of sorting sequences is indeed usually huge and having a complete representation of the space of solutions is desirable in order to obtain a more realistic study. Bergeron et al. [16] proposed a model to represent the universe of solutions in a compact way, grouping solutions into classes of equivalence, also called *traces*.

Two sequences of reversals are considered equivalent, and, consequently, are in the same trace, if one can be obtained from the other by a sequence of commutations of non-overlapping reversals (the operation of commutation can be applied to two reversals *ρ *and *θ *which appear consecutively in a sequence of reversals and consists in replacing the sequence *ρθ *by *θρ*). A trace is represented by its normal form [16,17], which corresponds to one of its sorting sequences that can be decomposed into substrings *s *= *u*_1 _< ⋯ <*u*_*m*_, such that:

• every pair of reversals of a substring *u*_*i *_is non-overlapping;

• for every reversal *ρ *of a substring *u*_*i *_(*i > *1), there is at least one reversal *θ *of the substring *u*_*i*-1 _such that *ρ *and *θ *overlap;

• every substring *u*_*i *_is increasing according to the lexicographic order.

Observe that in the original notation the normal form of a trace is *s *= *u*_1_|...|*u*_*m *_[16], but we prefer to use the symbol '<' instead of '|' as it gives a clearer indication of the order that applies between the substrings.

This method allows to reduce substantially the number of elements to be handled with respect to the whole set of solutions. The 28 sequences that sort the permutation (-3, 2, 1, -4), for instance, can be grouped in only two traces, one is {1}{1, 2, 3}{2}{4} (that contains 24 sequences), and the other is {1, 2, 4}{3} < {1, 3, 4} < {2, 3, 4} (that contains 4 sequences).

### Constructing traces

An algorithm to directly enumerate all the traces, computing the number of sequences in each trace, was given by Braga et al. [17], and consists in an incremental construction. At each iteration *i *the algorithm constructs the so called *i*-traces for the given permutations *π *and *π*_*T*_, that are the traces that contain all the optimal *i*-sequences for sorting *π *into *π*_*T*_. The *i*-traces are constructed from the previous (*i *- 1)-traces with the following procedure. For each previous (*i *- 1)-trace *T*, whose normal form is *f*, the algorithm obtains an intermediary permutation *π*_*f *_= *π *∘ *f*. Then it calculates all the next optimal 1-sequences for *π*_*f *_with the help of an algorithm proposed by Siepel [15] and constructs the next *i*-traces by adding each one of the returned 1-sequences to the previous (*i *- 1)-trace *T*. Initially, all the *i*-traces obtained from the (*i *- 1)-trace *T *have the same number of sorting sequences than *T*. Then the algorithm verifies whether, for each one of the new *i*-traces, there is an equivalent *i*-trace that is present in the list of already constructed *i*-traces. If this is the case, only one of the two equivalent *i*-traces is kept in the list, but the number of sequences in it is the sum of the sequences in the two equivalent *i*-traces. At the end, we have the final list of *d*-traces, where *d *is the reversal distance of (*π*, *π*_*T*_), and the number of sorting sequences in each *d*-trace.

### Constructing perfect traces

Traces have been analysed with respect to common intervals, and the following proposition has been proven by Braga et al. [17]:

**Proposition 1**. *Every trace of optimal solutions for sorting a signed permutation by reversals contains either only perfect solutions or no perfect solution (Braga et al. *[17]).

Due to this property, a trace that contains perfect sorting sequences is called a *perfect trace*. Because the perfect sorting sequences are symmetric, the perfect traces are also symmetric (if T is a perfect trace sorting *π *into *π*_*T*_, then *inv*(*T*) = { *inv*(*s*) | *s *∈ *T*} is a perfect trace sorting *π*_*T *_into *π*).

To compute the perfect traces, we need to introduce a few modifications to the original algorithm. We should first compute the initial irreducible common intervals between the two given permutations. Then, each time we compute the 1-sequences with Siepel's algorithm, we need to verify whether each one of the resulting 1-sequences breaks or not an irreducible common interval initially detected (the 1-sequences that break irreducible common intervals are simply discarded). At the end, we have only the perfect traces, if at least one perfect trace exists (otherwise we have an empty result).

### Constructing progressive perfect subtraces

In this work, we propose to analyse the space of all sorting sequences with respect to the progressive detection of common intervals. First we observe that, if we consider the progressive detection of common intervals, Proposition 1 does not hold anymore. Considering the permutation (-5, -2, -7, 4, -8, 3, 6, -1), for instance, the sequences of reversals {2,..., 5, 7, 8}, {3, 8}, {3, 4, 7}, {1,..., 8}, {2}, {4}, {2, 3, 4}, {2,..., 6} and {3, 8}, {3, 4, 7}, {2,..., 5, 7, 8}, {1,..., 8}, {2}, {4}, {2, 3, 4}, {2,..., 6} are in the same trace but, while the first preserves the progressively detected common intervals (as we can see in Figure 1(C)), the second does not (after applying the two first reversals, {3, 8} and {3, 4, 7}, we have the permutation (-5, -2, 3, -4, 7, 8, 6) with the common interval {6, 7, 8} which overlaps with the third reversal, {2,..., 5, 7, 8}). Thus, when we take the progressively detected common intervals in consideration, for each trace, only a subset of its sorting sequences is selected. We call this subset a *progressive perfect subtrace*. Consider a progressive perfect subtrace *t *of optimal sequences sorting a permutation *π*. Frequently the normal form of the trace *T *that contains *t *is not part of *t*. Due to this, when constructing progressive perfect subtraces, we also give at least one valid representative of each progressive perfect subtrace *t*, besides the normal form of the trace *T *that contains *t*. A progressive perfect subtrace *t *can be thus represented by a 2-tuple (*e, f*), where *e *is any sorting sequence in *t *and *f *is the normal form of the trace *T *that contains *t*. The normal form of the sorting sequence described in Figure 1(C) is *f *= {1,..., 8}{2, 4, 7}{6} < {2, 3, 6, 8}{4, 5, 7} < {3,..., 7}{3,..., 8}{4, 5, 6}, which is not a progressive perfect sequence and cannot be taken as a valid representative. However, the progressive perfect subtrace that contains the sorting sequence described in Figure 1(C) can be represented by the 2-tuple (*e, f*), where the valid progressive perfect representative *e *is {2, 4, 7}, {4, 5, 7}, {6}, {2, 3, 6, 8}, {1,..., 8}, {3,..., 7}, {3,..., 8}, {4, 5, 6}.

In addition, since the progressive perfect sorting sequences are asymmetric, the progressive perfect subtraces are also asymmetric, that is, the inverse of the progressive perfect sequences in a subtrace sorting a first permutation into a second are not necessarily progressive perfect sequences sorting the second permutation into the first.

To construct the progressive perfect subtraces, we need to modify the original algorithm of Braga et al. [17]. Analogously to the notation given by Braga et al., a progressive perfect subtrace whose sorting sequences have *i *reversals is called progressive perfect *i*-subtrace, and a progressive perfect *k*-subtrace *t' *is a *k*-*prefix *of a progressive perfect *i*-subtrace *t *(*k *≤ *i*) if each *k*-sequence of *t' *is a prefix of an *i*-sequence of *t*. To compute the progressive perfect subtraces, as in the original algorithm developed by Braga et al. [17], at each step we use the algorithm of Siepel [15] to list all possible 1-sequences. Then we filter these 1-sequences to discard those that break irreducible common intervals progressively detected. As a result of this procedure (see Algorithm 1), we construct directly the progressive perfect subtraces.

**Algorithm 1**: Enumerating all the progressive perfect subtraces of two signed permutations

**Input**: Two signed permutations *π *and *π*_*T*_

**Output**: The representative, normal form and counter (*e, f, c*) of each progressive perfect subtrace of sequences sorting *π *into *π*_*T*_

   *d ← *reversal distance of (*π*, *π*_*T*_)

   ← ∅

   *I*_0_ ← {*θ *| *θ *is an irred. comm. int. of *π *and *π*_*T *_}

   *S*_0 _← {*ρ *| *ρ *is an opt. 1-seq. for *π *→ *π*_*T *_} [*Siepel [15]*]

   **for each **1-seq. *ρ *∈ *S*_0 _**do **

      **if ***ρ *does not break an int. in *I*_0 _[*filter*] **then **

         insert (*ρ*, *ρ*, 1) in  [*each perf. first *1-*seq. is a prog. perf*. 1-*subtr*.]

      **end if**

   **end for**

   **for each **integer *i *from 2 to *d ***do**

       ← ∅ [*to keep all prog. perf. i-subtr*.]

      **for each **(*e, f, c*) in  [(*e, f*) *is a prog*. *p*. (*i *- 1)-*subtr*.; *c is the counter*] **do**

         *π*_*f*_ ← *π *∘ *f *[*apply the *(*i *- 1)-*seq*. *f to π *]

         *I*_*f*_ ← {*θ *| *θ *is an irred. comm. int. of *π*_*f *_and *π*_*T *_}

         *S*_*f*_ ← { *ρ *| *ρ *is an opt. 1-seq. for *π*_*f *_→ *π*_*T *_} [*Siepel [15]*]

         **for each **1-seq. *ρ*∈ *S*_*f *_**do**

            **if ***ρ *does not break an int. in *I*_*f *_[*filter*] **then **

               *f_ρ_*← *f *+ *ρ *[*add ρ to extend f; see Braga et al. [17]*]

               **if **there exists (*e'*, *f'*, *c*') ∈  such that *f' *= *f_ρ _*[***CMP***] **then **

                  *c' *← *c' *+ *c *[*update the counter*

                  *of the prog. perf. i-subtr*. (*e'*, *f'*)]

               **else**

                  *e_ρ_*← *e *· *ρ *[*concat. ρ to the seq. e*]

                  insert (*e_ρ _*, *f_ρ _*, *c*) in  [(*e_ρ _*, *f_ρ_*) *is a*

                  *prog. perf. i-subtr.; c is the counter*]

               **end if**

            **end if**

         **end for**

      **end for**

       ← 

   **end for**

   **return ** [*is the final set of progressive perfect d-subtraces sorting π into π_T_*]

As in the original algorithm, we may need to compare subtraces to verify whether a new subtrace *t *is present in the list of already constructed subtraces (Algorithm 1, step *CMP*). In order to do that, we use the normal form *f *of the trace *T *that contains *t*, and compare *f *to the normal forms of the traces that contain the already constructed subtraces. The normal form of an *i*-trace is constructed incrementally, from the normal form of one of its (*i *- 1)-prefixes [17]. The representative of an *i*-subtrace is also constructed incrementally, by concatenating a reversal to the end of the sequence that represents one of its (*i *- 1)-prefixes. Thus, for two given permutations *π *and *π*_*T*_, at the end of Algorithm 1, we have the list of all non-empty progressive perfect subtraces and each progressive perfect subtrace *t *is represented by a 2-tuple (*e, f*), where *e *is any progressive perfect sorting sequence in *t *and *f *is the normal form of the trace *T *that contains *t*. If no progressive perfect sequence exists for sorting *π *into *π*_*T*_, we have an empty result.

## Results and discussion

### Theoretical complexity and experimental results

The original algorithm of Braga et al. [17] has complexity *O*(), where *n *is the size of the input permutation *π*, *N *is the number of computed final traces and *k*_*max *_is the maximum value for the *width *of a final trace [17]. The 4 in the exponent of this formula is due to the processing of each (*i *- 1)-trace *T *to generate the subsequent *i*-traces, given by the following procedure: (1) apply the sequences of reversals of *f*, which is the normal form of *T*, on the initial permutation *π *to obtain *π*_*f *_; (2) run Siepel's algorithm [15] over *π*_*f *_; (3) add each one of the *O*(*n*^2^) reversals returned by Siepel's algorithm to *T *to build a new *i*-trace. The complexity of this procedure is (1) + (2) + (3) = *n*^2 ^+ *n*^3 ^+ *n*^2^.*n*^2^, that results in *O*(*n*^4^).

With respect to the original algorithm, we added two new steps to the processing of an (*i *- 1)-subtrace *t *to generate the following *i*-subtraces: (1B) computing the irreducible common intervals in *π*_*f *_; (2B) filtering each reversal returned by Siepel's algorithm. Computing the irreducible common intervals can be done in *O*(*n*) time [21]. Filtering the reversals, that is, testing whether each one of the *O*(*n*^2^) reversals returned by Siepel's algorithm overlaps with each one of the irreducible common intervals can take *n*^2^.*n.n*, because comparing two intervals (a reversal and a common interval) takes *O*(*n*) and each reversal has to be compared to *O*(*n*) [21] irreducible common intervals. Thus, the complexity of processing an (*i *- 1)-subtrace is given by (1) + (1*B*) + (2) + (2*B*) + (3) = *n*^2 ^+ *n *+ *n*^3 ^+ *n*^4 ^+ *n*^4^, that results in *O*(*n*^4^). Consequently, the complexity of the modified algorithm is *O*(), where *L *is *O*(*N*) and represents the number of computed final progressive perfect subtraces.

Observe that, to calculate perfect traces, we compute the irreducible common intervals once for the input permutation *p*, and then we only have to introduce the filtering step, whose complexity is *O*(*n*^4^), in the original algorithm. Thus, the theoretical complexity in this case is *O*(), where *M*, the number of computed final perfect traces, is also *O*(*N*).

We implemented both algorithms, to compute perfect traces and progressive perfect subtraces, integrated to the BAOBABLUNA package [22], which had already the implementation of computing traces and is available online at http://pbil.univ-lyon1.fr/software/luna/. Although the theoretical complexity of the new approaches is equal to the original approach, the experimental results, presented in Table 1, revealed that searching for reversals that do not break common intervals is a constraint that usually reduces the number of traces and solutions, and consequently, the execution time. Moreover, the reduction is considerably higher when we apply the progressive detection of common intervals (usually *L *<*M *<<*N*).

**Table 1:** Experimental results

Perm.	Algorithm	N_*S*_	N_*T*_	Exec. time
*A*, ℐ_8_ *d *= 8	all (*A *↔ ℐ_8_)	81, 869	377	≃ 5 s
	prf(*A *↔ ℐ_8_)	51,304	92	≃ 5 s
	prg (*A *→ ℐ_8_)	11, 568	12	≃ 3 s
	prg (ℐ_8 _→ *A*)	8, 400	5	≃ 2 s

*B*, ℐ_16_ *d *= 12	all (*B *↔ ℐ_16_)	505, 634, 256	21, 902	≃ 7.3 m
	prf (*B *↔ ℐ_16_)	122, 862, 960	171	≃ 27 s
	prg (*B *→ ℐ_16_)	5, 963, 760	6	≃ 14 s
	prg (ℐ_16_→ *B*)	5, 393, 520	9	≃ 16 s

The experimental results of computing traces (all), perfect traces (prf) and progressive perfect subtraces (prg; in both directions), considering the pairs of permutations (*A*, ℐ_8_) and (*B*, ℐ_16_), where *A *= (-5, -2, -7, 4, -8, 3, 6, -1) and *B *= (-12, 11, -10, -1, 16, -4, -3, 15, -14, 9, -8, -7, -2, -13, 5, -6).

The columns N_*S *_and N_*T *_give, respectively, the resulting number of sorting sequences and traces for each approach. All algorithms are part of the BAOBABLUNA package [22]. Experiments were made on a 64 bit personal computer with two 3GHz CPUs and 2GB of RAM and the execution time is given in seconds (s) or minutes (m).

### Accepting common interval breaks

As mentioned, searching for perfect traces or for progressive perfect subtraces may reduce the number of sorting sequences and traces. However, there is no guarantee that these constrained traces exist, thus those approaches may eventually lead to empty results. For example, the permutation (1, 3, -2, -11, 5, -9, -10, 8, 6, -7, -4, 12), whose reversal distance is 9, has no perfect sorting sequence and no progressive perfect sorting sequence. Due to this, Braga et al. [17] proposed the construction of near-perfect traces, accepting a bounded number of breaking reversals per trace. In their approach, a reversal can have a score of 0 if it does not break any common interval, or a score of 1 if it breaks one or more common intervals. The score of a sequence of reversals is bounded by *k*, that is, each sorting sequence in a near-perfect trace has at most *k *breaking reversals.

The consequences of accepting common interval breaks have not been largely discussed by Braga et al. It was particularly not mentioned that, differently from the perfect sequences, the near-perfect sequences of reversals are asymmetric, that is, inverting a near-perfect sequence of reversals sorting a permutation *π *into *π*_*T *_with score equal to *k *does not necessarily result in a near-perfect sequence of reversals sorting *π*_*T *_into *π *with the same score *k*. The reason is that, after being broken, a common interval is no longer common and should be removed from the initial list of common intervals. Thus, the list of common intervals may be different at each step and depends on the order in which the reversals are applied. For example, considering the permutation *p *= (1, 3, -2, -11, 5, -9, -10, 8, 6, -7, -4, 12), there is no perfect sequence of reversals sorting *π *into ℐ_12_, and we must accept at least two breaking reversals. The irreducible common intervals between these two permutations are {1, 2, 3}, {2, 3}, {2,..., 11}, {2,..., 12}, {4,..., 10}, {4,..., 11}, {4,..., 12}, {5,..., 10}, {5,..., 11}, {5,..., 12}, {5,..., 11}, {5,..., 12}, {6, 7}, {6, 7, 8}, {6, 7}, {8, 9, 10}, {9, 10}. To construct a sequence of score 2, we can first apply the non-breaking reversals {2, 3}, {3}, {4,..., 11}, {5,..., 10}, {7} and {9} and obtain (1, 2, 3, 4, 5, 9, -10, 8, 6, 7, 11, 12). Then we apply the reversal {6, 7, 8, 10} that breaks the intervals {8, 9, 10} and {9, 10}. The next reversal is {6, 7, 9}, that breaks the interval {6, 7, 8}. Then the last reversal is {8, 9}, which is non breaking. Observe however that if we do not remove the already broken intervals from the initial list, the last reversal should be considered a breaking one (it also "breaks" the interval {9, 10}), and this sequence would have a score of 3 instead of 2. A consequence of updating the list of common intervals when we accept a number of interval breaks bounded by *k *is that we have near-perfect subtraces instead of traces. Similar to what happens when we use a progressive detection, only a subset of the sequences in a trace may achieve the given score *k*, and this process is not symmetric. Thus, when we accept interval breaks, we are not able to keep the symmetry. In other words, although the perfect traces are symmetric, the near-perfect subtraces are asymmetric and this should be taken in consideration when we apply this method to the analysis of real cases.

We can also accept interval breaks when searching for progressive perfect subtraces. As for the progressive perfect subtraces, the progressive near-perfect subtraces are also asymmetric. Nevertheless, we may use a different score system. In our model, a reversal can have a score of 0 if it does not break any common interval, or a score of 1 if one of its extremities breaks common intervals, or of 2 if both extremities break common intervals. The score of a sequence of reversals is still given by the sum of the scores of its reversals.

### Reconstructing the evolutionary scenario of *Rickettsia felis*

**Figure 2:**
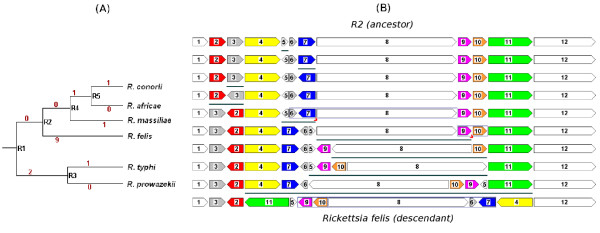
**Evolutionary scenario between *Rickettsia felis *and one of its ancestors. (A) **Phylogenetic tree of six *Rickettsia *(extracted from [2]). The numbers on the edges give the reversal distance between the genomes on the vertices, which could be either a current species or an ancestor (*R*1, *R*2, *R*3, *R*4 and *R*5). **(B) **The optimal sequence of reversals to transform the ancestor *R*2 into *Rickettsia felis *(proposed by Blanc et al. [2] with the help of the software GRIMM[10]). The two common interval breaks are indicated by the "comma" signs.

We used our approach of searching for progressive perfect subtraces to analyse the evolutionary scenario between the bacterium *Rickettsia felis *and one of its ancestors. The *Rickettsia *bacteria are intracellular parasites. There are several completely sequenced *Rickettsia *genomes, and most of them are closely related. The evolutionary scenario of six *Rickettsia *species was recently analysed and the ancestors *R*1, *R*2, *R*3, *R*4 and *R*5 (represented in Figure 2(A)) were reconstructed [2]. In particular, one optimal sequence of reversals, obtained by Blanc et al. [2] with the help of the software GRIMM[10], was proposed to transform *R*2 into *Rickettsia felis *(see Figure 2(B)).

In order to be able to use the asymmetric progressive perfect approach, we analyse the space of solutions from the descendant (*Rickettsia felis*) to the ancestor (*R*2). Those genomes have 12 blocks of contiguous homologous genes, mapped as ℐ_12 _for *R*2, and the permutation (1, 3, -2, -11, 5, -9, -10, 8, 6, -7, -4, 12) for *R. felis *(see Figure 2(B)). The reversal distance between these two genomes is equal to 9, and the complete analysis of the traces of sequences sorting *R. felis *into *R*2 resulted in 546840 sorting sequences, distributed in 13 traces (Table 2).

**Table 2:** Traces of sequences sorting *R. felis *into its ancestor

Trace	Trace normal form (f)	**# seq**.	**# seq**.
	Subtrace representative (e)	trace	subtr.
1.	*f *= {2, 3}{3}{4,..., 11}{5}{5, 8, 9, 10}{7}{8, 10} < {5, 6, 7}{8, 9}	90720	45360
	*e *= {4,..., 11}{5, 8, 9, 10}{8, 10}{8, 9}{5, 6, 7}{2, 3}{3}{7}{5}		
2.	*f *= {2, 3}{3}{4,..., 11}{5,..., 10}{6}{6, 7, 8, 10}{6, 8} < {6,..., 9}{7, 8}	90720	45360
	*e *= {2, 3}{3}{4,..., 11}{5,..., 10}{6}{6, 7, 8, 10}{6, 8}{6,..., 9}{7, 8}		
3.	*f *= {2, 3}{3}{4,..., 11}{5,..., 10}{6}{6, 8, 9, 10}{8, 10} < {7,..., 10}{8, 9}	90720	45360
	*e *= {2, 3}{3}{4,..., 11}{5,..., 10}{6}{6, 8, 9, 10}{7,..., 10}{8, 10}{8, 9}		
4.	*f *= {2, 3}{3}{4,..., 11}{5,..., 10}{6, 7, 8, 10}{7}{9} < {6, 7, 9} < {8, 9}	60480	60480
	*e *= {2, 3}{3}{4,..., 11}{5,..., 10}{6, 7, 8, 10}{7}{9}{6, 7, 9}{8, 9}		
5.	*f *= {2, 3}{3}{4,..., 11}{5,..., 10}{6, 8}{9}{10} < {6, 9, 10} < {7,..., 10}	60480	0
6.	*f *= {2, 3}{3}{4,..., 11}{5,..., 10}{7}{8, 10}{10} < {6, 7, 10} < {6,..., 9}	60480	0
7.	*f *= {2, 3}{3}{4,..., 11}{5, 9, 10}{7}{9}{10} < {5, 8} < {5, 6, 7}	60480	60480
	*e *= {2, 3}{3}{4,..., 11}{5, 9, 10}{7}{9}{10}{5, 8}{5, 6, 7}		
8.	*f *= {2, 3}{3}{4,..., 11}{5, 8, 9, 10}{5, 9, 10}{7} < {5, 6, 7, 9, 10} < {6, 7, 8, 10} < {6,..., 9}	9072	0
9.	*f *= {2, 3}{3}{4,..., 11}{5, 8, 9, 10}{6}{8, 10} < {5, 6, 8, 9} < {5, 7, 8, 9} < {6,..., 9}	6048	0
10.	*f *= {2, 3}{3}{4,..., 11}{5, 9, 10}{6, 8}{10} < {5, 6, 9} < {5, 7, 8, 9} < {6,..., 9}	6048	0
11.	*f *= {2, 3}{3}{4,..., 11}{6}{6, 8, 9, 10} < {5, 6, 8, 10} < {5, 6, 8, 9} < {5,..., 8}{7, 8}	6048	6048
	*e *= {2, 3}{3}{4,..., 11}{6}{6, 8, 9, 10}{5, 6, 8, 10}{5, 6, 8, 9}{5,..., 8}{7, 8}		
12.	*f *= {2, 3}{3}{4,..., 11}{5}{6, 8, 9, 10} < {5, 6, 8, 10} < {5, 7, 9} < {6, 7, 9} < {8, 9}	3024	0
13.	*f *= {2, 3}{3}{4,..., 11}{6, 8} < {6, 9, 10}{7, 8} < {5, 6, 10} < {5, 6, 9} < {5,..., 8}	2520	0

	Total	546840	263088

The 546840 sequences that sort *Rfe *= (1, 3, -2, -11, 5, -9, -10, 8, 6, -7, -4, 12) (*R. felis*) into *R*2 = (1, 2, 3, 4, 5, 6, 7, 8, 9, 10, 11, 12) are distributed in 13 traces. Each trace is represented by its normal form. The third column indicates the number of sequences in each trace. When we apply the progressive detection of common intervals, accepting at most two common interval breaks, we obtain 263088 sequences distributed in 6 progressive near-perfect subtraces (subsets of traces 1, 2, 3, 4, 7 and 11). Each progressive near-perfect subtrace is represented by a 2-tuple (*e *is the subtrace representative, *f *is the trace normal form). The fourth column gives the number of sequences in each subtrace.

**Figure 3:**
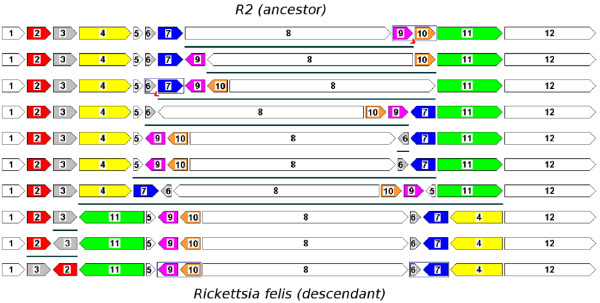
**Alternative scenario between *Rickettsia felis *and one of its ancestors**. An alternative optimal sequence of reversals to transform the ancestor *R*2 into *Rickettsia felis*, that is the inverse of a sequence taken from subtrace 3 of Table 2. The two common interval breaks are indicated by the "comma" signs.

We then analysed the universe of solutions between *Rickettsia felis *and *R*2 taking into account the progressively detected common intervals. We had to relax the constraint to accept two interval breaks, because the result of searching for progressive perfect subtraces that do not break any common interval or that break one common interval per sorting sequence is empty. Accepting two interval breaks per sorting sequence, more than half of the solutions and traces from the complete solution space is discarded (see the results in Table 2). We observed that the scenario proposed in [2] (Figure 2(B)) was selected by the construction of progressive near-perfect subtraces accepting two common interval breaks per solution (it is the inverse of a sequence in subtrace 1 of Table 2). However, there are still many other possibilities that have the same score with respect to progressively detected common interval breaks. We can, for instance, take an alternative sequence from subtrace 3 in Table 2 (Figure 3).

### Final remarks

In this work we introduced a new approach to explore the universe of optimal sequences of reversals sorting a genome into another, that consists in preserving the common intervals progressively detected between the two analysed genomes. We adapted an algorithm given by Braga et al. [17], showing that, with the same theoretical complexity of the original algorithm, we can obtain all the classes of equivalent sequences of reversals that preserve entirely the common intervals progressively detected. Since we select directly the sequences that respect this constraint, our approach achieves a significative reduction of the universe of solutions and may be able to deal with more distant genomes than the original algorithm.

We showed that this approach is asymmetric, because for two given genomes *A *and *B*, the results of the analysis of the sequences sorting *A *into *B *can not be obtained from the results of the analysis of the sequences sorting *B *into *A*. However, this may be biologically valuable when the analysed genomes have a clear relation ancestor-descendant. The analysis may be done from the descendant to the ancestor, as the objective is to regroup clusters of genes that existed in a past time. In other words, from the ancestor to the descendant the clusters were progressively broken.

We applied our method to analyse the evolutionary scenario between the bacterium *Rickettsia felis *and one of its ancestors, that was reconstructed by Blanc et al. [2]. We showed that our approach is able to provide a more complete analysis of the different possibilities of transforming the ancestor into *Rickettsia felis *by reversals, instead of taking an arbitrary sequence of reversals to explain a scenario, as was done by Blanc et al. In particular, we showed that the scenario proposed by Blanc et al. achieves the maximum score according to our method, but that there are also several other scenarios that achieve such maximum score.

## Competing interests

The authors declare that they have no competing interests.

## Authors' contributions

This work was conceived as a result of studies carried out by all three authors. MDVB did the implementation and performed the tests. All authors participated in the analysis of the results, and in editing the manuscript that was first drafted by MDVB. All authors read and approved the final manuscript.
